# High‐Security X‐Ray Imaging Encryption Based on Irreversible Acid‐Responsive Radioluminescence Memory Scintillator

**DOI:** 10.1002/advs.202520158

**Published:** 2025-12-17

**Authors:** Lin Liu, Shanshan Peng, Hanjing Guo, Linping He, Xiaofang Luo, Zixuan Li, Lisen Lin, Huanghao Yang

**Affiliations:** ^1^ Department of Nuclear Medicine the First Affiliated Hospital Fujian Medical University Fuzhou 350005 China; ^2^ New Cornerstone Science Laboratory MOE Key Laboratory for Analytical Science of Food Safety and Biology College of Chemistry Fuzhou University Fuzhou 350108 China; ^3^ Department of Nuclear Medicine, National Regional Medical Center, Binhai Campus of the First Affiliated Hospital Fujian Medical University Fuzhou 350212 China

**Keywords:** acid‐responsiveness, high‐security, persistent radioluminescence, radioluminescence memory scintillator, X‐ray imaging encryption

## Abstract

X‐ray imaging encryption has garnered significant attention but remains vulnerable to information leakage and hard to detect breaches. Herein, an irreversible acid‐responsive radioluminescence memory scintillator, Zn_1.3_Ga_1.4_Ge_0.3_O_4_:Cr^3+^@Zn_2_GeO_4_:Eu^3+^ (ZGGO@ZGO), is developed, which not only enhances information security but also enables real‐time leakage detection. The ZGGO@ZGO exhibits visible and near‐infrared (NIR) dual‐emitting persistent radioluminescence (PRL), in which the visual signal acts as a decoy to mislead potential offenders. Upon acid treatment, Zn_2_GeO_4_:Eu^3+^ (ZGO) shell undergoes rapid degradation, enabling ZGGO@ZGO to realize the transformation of dual‐emitting PRL into single‐emitting imperceptible NIR PRL. The transformation of PRL effectively prevents visual detection by the naked eye, allowing decryption only through a charge‐coupled device (CCD) camera to reveal the hidden information. Through engineering a dual‐layer security system composed of ZGGO@ZGO and ZGO, visual misdirection is synergized with sequential logic gates requiring acid treatment and CCD detection, significantly reducing leakage risks. Importantly, the acid‐responsive PRL transformation is irreversible. Once decoded, the information is permanently locked in its altered state, allowing the detection of information leakage and the effective mitigation of subsequent harm. This approach surpasses traditional encryption methods by providing both enhanced security and the ability to identify unauthorized access, potentially revolutionizing information protection in sensitive applications.

## Graphical abstract

An irreversible acid‐responsive radioluminescence memory scintillator, Zn_1.3_Ga_1.4_Ge_0.3_O_4_:Cr^3+^@Zn_2_GeO_4_:Eu^3+^ (ZGGO@ZGO), is developed in this work. The engineered dual‐layer security system composed of ZGGO@ZGO and ZGO achieves a combined effect of visual misdirection and sequential logic gates requiring acid treatment and CCD detection, significantly reducing leakage risks while enabling the detection of information breaches.

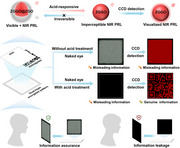

## 1 Introduction

Scintillators capable of converting X‐ray into visible light have been developed since Rontgen discovered X‐ray in 1885.^[^ ^1^, ^2^ ^]^ These materials play a pivotal role in modern X‐ray imaging technologies, where the optical signals they generate are captured and converted into visual information or electrical signals for various applications such as CT imaging, industrial flaw detection, security screening, and so on.^[^ ^3^, ^4^, ^5^, ^6^ ^]^ However, traditional X‐ray imaging technologies often rely on the integration of flat‐panel detectors with scintillators as the core component, which are hindered by inherent limitations such as light scattering, optical crosstalk, and image distortion.^[^ ^7^, ^8^ ^]^ Currently, significant efforts have been directed toward the development of flexible scintillator films, which offer outstanding X‐ray imaging performance due to their high flexibility, enabling them to conform to the contours of objects and thereby mitigate the drawbacks of traditional flat‐panel detectors.^[^ ^9^, ^10^, ^11^, ^12^ ^]^ Despite remarkable advancements in the fabrication and application of flexible scintillator films, several challenges persist. One of the most significant hurdles lies in its real‐time luminescence, a feature that is necessary to collect optical signals during X‐ray stimulation process,^[^ ^13^ ^]^ typically generating inconvenient equipment setups and restricting practical application scenarios. Ongoing research aims to overcome this issue, paving the way for more versatile and efficient X‐ray imaging technologies.

Radioluminescence memory scintillators uniquely store excitation energy and release photon emissions over extended periods ranging from seconds to days.^[^ ^14^, ^15^, ^16^, ^17^ ^]^ This property, which is different from that of conventional scintillators, offers significant potential for advancing X‐ray imaging technologies. Recently, we pioneered a flat‐panel‐free X‐ray luminescence extension imaging using radioluminescence memory scintillators, leveraging their photophysical characteristics of excitation and emission separation.^[^ ^18^ ^]^ This innovation not only simplifies the X‐ray imaging system but also enables visualization of X‐ray images after irradiation has ceased, garnering attention in X‐ray imaging encryption.^[^ ^19^, ^20^, ^21^, ^22^, ^23^ ^]^ However, a limitation of existing radioluminescence memory scintillators is that their persistent radioluminescence (PRL) primarily falls within the visible spectrum, making the information susceptible to trial‐and‐error attacks with naked‐eye; More critically, once the PRL reverts to its initial state (disappearance of PRL) following decryption, it becomes impossible to determine whether the encrypted information has been accessed.^[^ ^24^, ^25^, ^26^ ^]^ This inability to detect information breaches and promptly update encrypted information poses significant risks, particularly in areas impacting public livelihood and national security. Unfortunately, to date, there is no report on the development of a high‐security X‐ray imaging encryption scheme capable of both non‐naked‐eye decryption and information leakage detection.

**Scheme 1:**
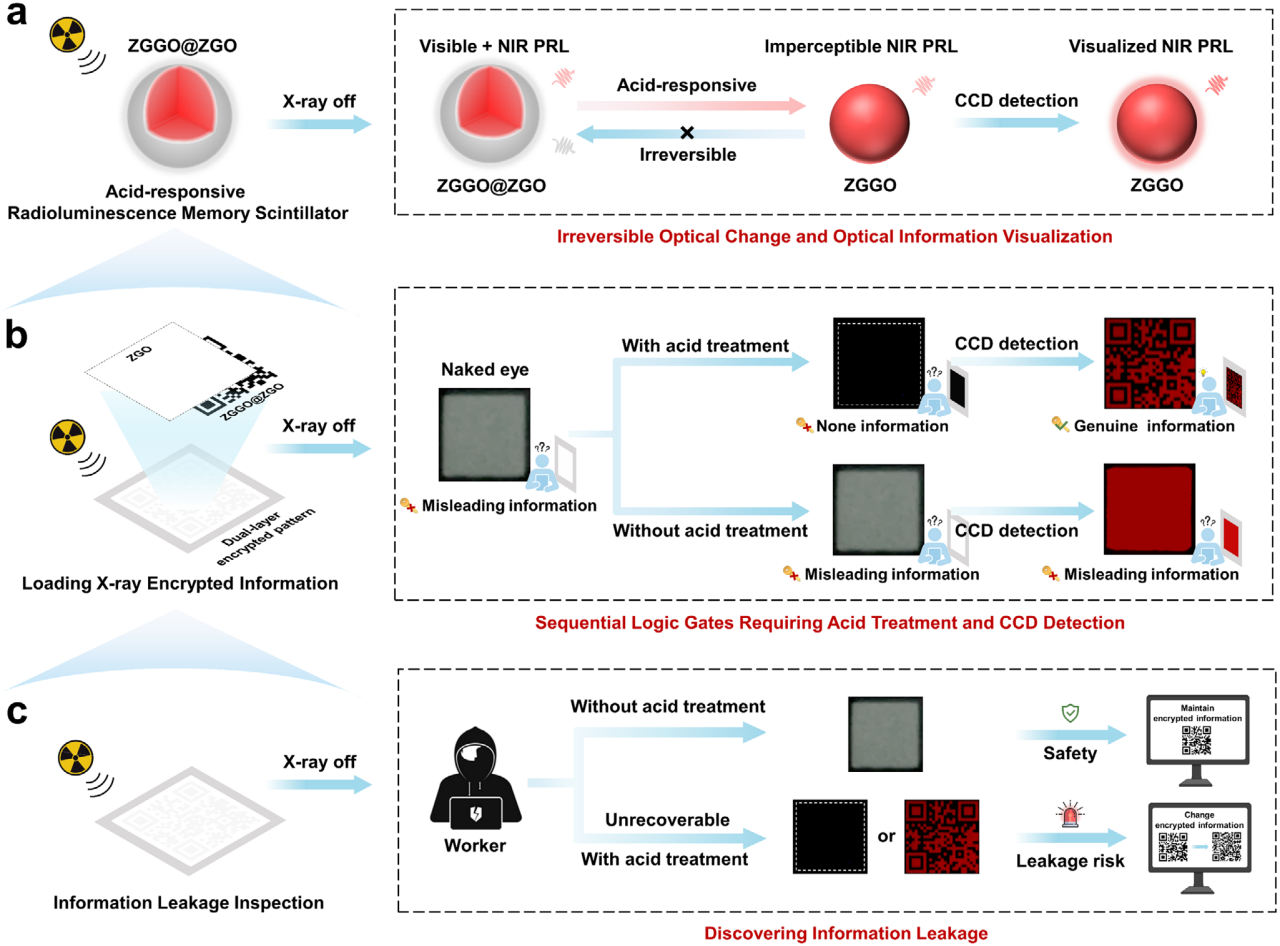
Schematic illustration of high‐security X‐ray imaging encryption. a) The irreversible acid‐responsive ability of ZGGO@ZGO. b) High‐security decryption scheme requiring acid treatment and CDD detection sequential logic gates based on a dual‐layer security system of ZGGO@ZGO and ZGO. c) Discovering information leakage based on the irreversible acid‐responsive degradability.

To address these challenges, we designed an irreversible acid‐responsive radioluminescence memory scintillator, Zn_1.3_Ga_1.4_Ge_0.3_O_4_:Cr^3+^@Zn_2_GeO_4_:Eu^3+^ (ZGGO@ZGO), which enables non‐visual decryption and information leakage detection for high‐security X‐ray imaging encryption (**Scheme** **1**). Specifically, ZGGO@ZGO demonstrates visible and near‐infrared (NIR) dual‐emitting PRL after X‐ray irradiation. The visible light component makes the optical signal detectable to the naked eye, serving as misleading information. After acid treatment, the original dual‐emitting PRL is converted into a single‐emitting NIR PRL that is imperceptible to the naked eye but can be decrypted using a charge‐coupled device (CCD) camera, serving as genuine information (Scheme 1a). Integration of ZGGO@ZGO and ZGO into a dual‐layer security system enables a sophisticated encryption strategy that combines visual deception with sequential logic gates requiring acid treatment and CCD detection, thereby substantially reducing the risk of information leakage (Scheme 1b). More importantly, the acid‐responsive PRL transformation behavior of ZGGO@ZGO is irreversible. Once decoded, the single‐emitting NIR PRL cannot revert to its initial dual‐emitting state, which enables timely detection of information leakage by observing whether the visual information remains after X‐ray irradiation, a capability unattainable with conventional encryption methods (Scheme 1c). In summary, the unique acid‐responsive properties of ZGGO@ZGO offer a robust and secure solution for X‐ray imaging encryption.

## 2 Results and Discussion

### 2.1 Design and Synthesis of ZGGO@ZGO

**Figure 1:**
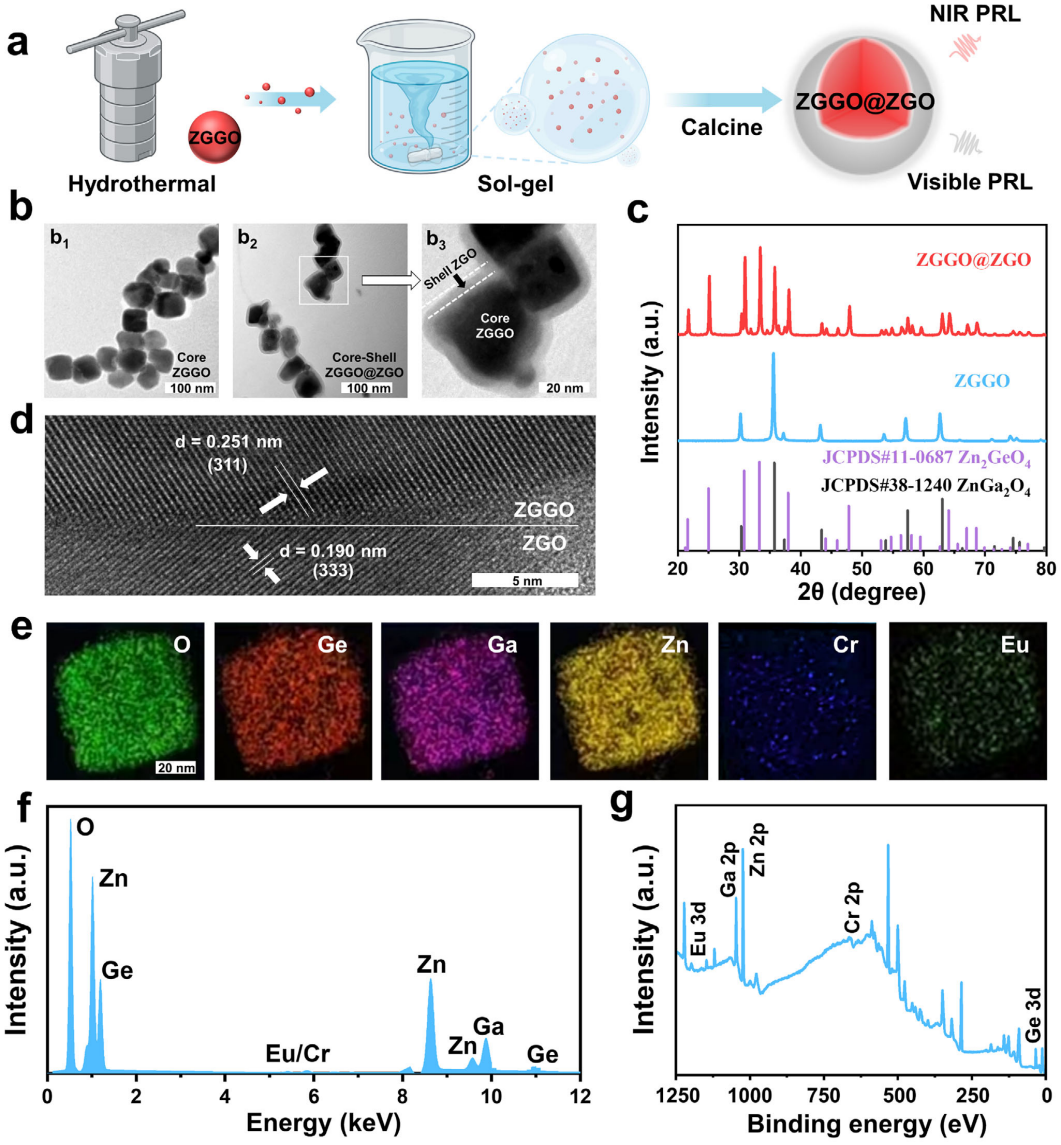
The synthesis and characterization of ZGGO@ZGO. a) Schematic diagram illustrating the preparation process of ZGGO@ZGO. b) TEM images of ZGGO and ZGGO@ZGO. c) XRD patterns of ZGGO and ZGGO@ZGO. d) HR‐TEM of ZGGO@ZGO. e) Element mapping images of ZGGO@ZGO. f) EDS and g) XPS spectrum of ZGGO@ZGO.

In this work, Zn_1.3_Ga_1.4_Ge_0.3_O_4_:Cr^3+^ (ZGGO) was synthesized as the core, and then a layer of Zn_2_GeO_4_:Eu^3+^ (ZGO) was in situ grown on its surface to fabricate the core‐shell structured ZGGO@ZGO nano‐system, as illustrated in **Figure** **1**a. First, a series of Cr^3+^‐doped Zn_1+x_Ga_2‐2x_Ge_x_O_4_ was synthesized. Zn_1+x_Ga_2‐2x_Ge_x_O_4_:Cr^3+^ (x = 0–0.4) exhibited the same crystal structure but varied in particle size and near‐infrared (NIR) persistent radioluminescence (PRL) intensity (Figures S1–S4, Supporting Information). Among them, ZGGO (x = 0.3) demonstrated optimal NIR PRL performance and acid‐stability (Figures S3–S5, Supporting Information). The PRL from ZGGO, emitting in the imperceptible NIR region, remained invisible to the naked eye yet was readily detectable using a NIR charge‐coupled device (CCD) camera (Figure S6, Supporting Information), thereby establishing its inherent non‐naked‐eye‐detectable trait. When Ga is completely substituted by Ge (X = 1, Zn_2_GeO_4_), the crystal structure and morphology changed, and the PRL also shifted from NIR to the visible region (Figures S7–S9, Supporting Information). Furthermore, the visible PRL intensity of Zn_2_GeO_4_ was enhanced by doping with Eu^3+^ (ZGO, Figure S10, Supporting Information). A strong acid, capable of reacting with a salt of a weak acid, can yield the corresponding weak acid and a salt of the strong acid. Therefore, acids stronger than H_4_GeO_4_ (such as acetic acid) could displace the ${\mathrm{GeO}}_{4}^{4-}$ in Zn_2_GeO_4_, leading to the degradation of ZGO and the quenching of its PRL (Figure S11, Supporting Information).^[^ ^27^ ^]^ Consequently, the core‐shell structured ZGGO@ZGO nano‐system can be used as an irreversible acid‐responsive radioluminescence memory scintillator with PRL‐changing capabilities: before acid treatment, ZGGO@ZGO exhibits dual‐emitting PRL in both the visible and NIR regions; after acid treatment, the dual‐emitting PRL of ZGGO@ZGO irreversibly transforms into an invisible single‐emitting NIR PRL, which is expected to enable non‐visual decryption and leakage detection for high‐security X‐ray imaging encryption.

To confirm the successful synthesis of ZGGO@ZGO, transmission electron microscopy (TEM) images were obtained (Figure 1b). The ZGGO core exhibited a diameter of ≈50 nm (Figure 1b_1_). After in situ growth of ZGO on the ZGGO surface, the core‐shell structure was clearly observed (Figure 1b_2_,b_3_). X‐ray diffraction (XRD) patterns of ZGGO@ZGO were then analyzed (Figure 1c). The diffraction peaks of ZGGO@ZGO closely matched the combination of Zn_2_GeO_4_ (standard card: JCPDS#11‐0687) and ZnGa_2_O_4_ (standard card: JCPDS#38‐1240), confirming its composite structure. This result was further supported by high‐resolution TEM (HR‐TEM, Figure 1d). The crystalline structure of ZGGO@ZGO displayed lattice spacings of 0.25 nm (corresponding to the spacing of the (311) lattice planes of ZnGa_2_O_4_, standard card: JCPDS#38‐1240) and 0.19 nm (corresponding to the spacing of the (333) lattice planes of Zn_2_GeO_4_, standard card: JCPDS#11‐0687), which were attributed to the ZGGO core and ZGO shell, respectively. Additionally, element mapping images (Figure 1e), energy‐dispersive X‐ray spectroscopy (EDS, Figure 1f), X‐ray photoelectron spectroscopy (XPS, Figure 1g), and diffuse reflectance spectroscopy (DRS, Figure S12, Supporting Information) were conducted. These results collectively verify the successful preparation of ZGGO@ZGO.

### 2.2 Photophysical Properties of ZGGO@ZGO

**Figure 2:**
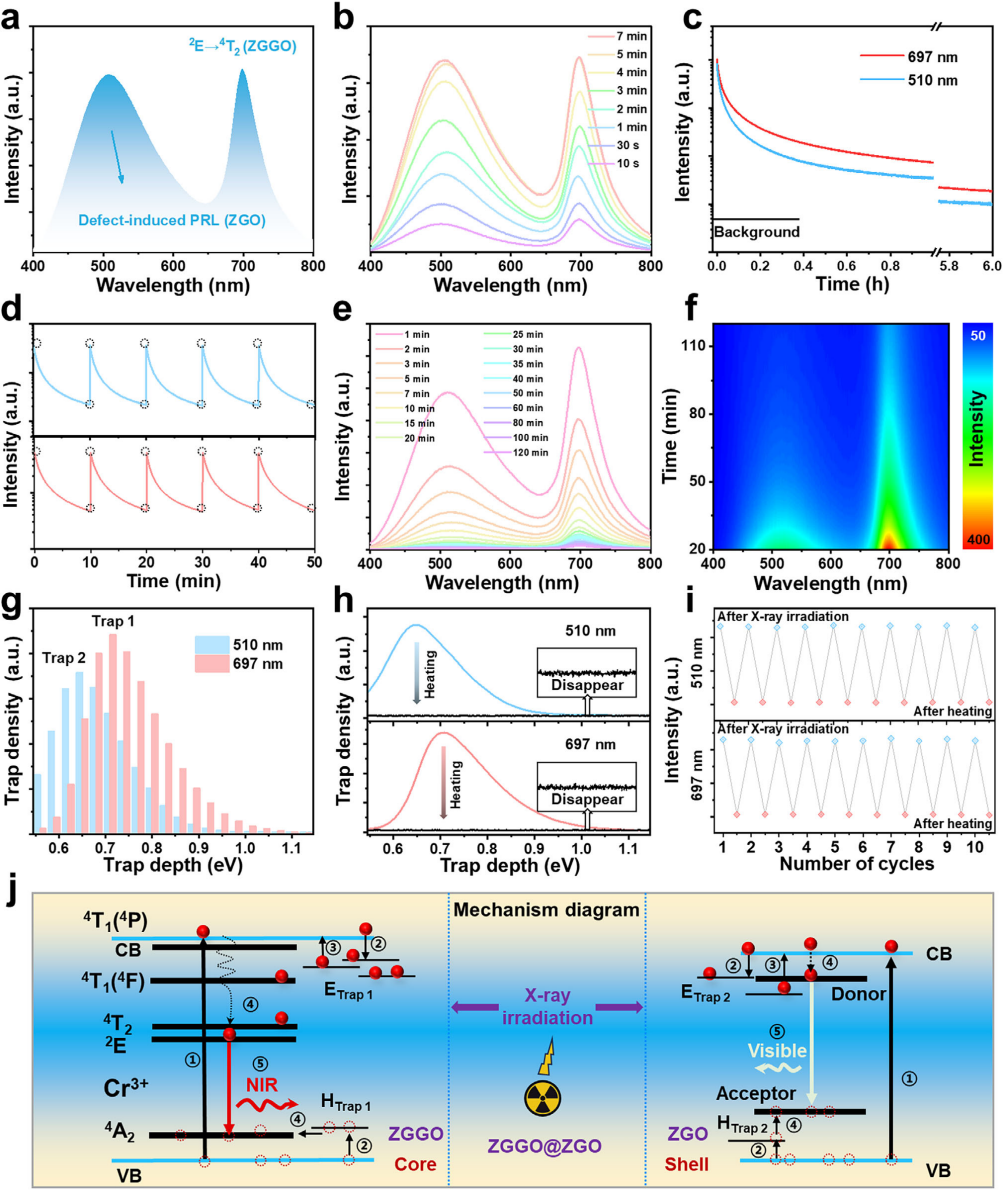
The photophysical properties of ZGGO@ZGO. a) PRL spectrum of ZGGO@ZGO after 5 min of X‐ray irradiation. b) PRL spectra of ZGGO@ZGO after varying durations of X‐ray irradiation. (c) PRL decay curve of ZGGO@ZGO following 5 min of X‐ray irradiation. d) Repetitive PRL decay profiles of ZGGO@ZGO after 5 min of X‐ray irradiation. e) PRL spectra of ZGGO@ZGO at different decay times after X‐ray irradiation. f) Contour maps depicting PRL intensity as a function of decay time and emission wavelength for ZGGO@ZGO after X‐ray irradiation. g) Trap distribution of ZGGO@ZGO after 5 min of X‐ray irradiation. h) Changes in trap distribution of ZGGO@ZGO after heating at 150 °C for 10 min. i) PRL intensity at 510 nm (visible region) and 697 nm (NIR region) as a function of alternating cycles of X‐ray irradiation (5 min) and heating (150 °C for 10 min). j) Proposed PRL mechanism of ZGGO@ZGO.

After confirming the successful synthesis of ZGGO@ZGO, we investigated its photophysical properties. First, the persistent radioluminescence (PRL) spectrum was recorded after 5 min of X‐ray irradiation (**Figure** **2**a). Two emission bands were observed at 510 nm (visible region) and 697 nm (NIR region), consistent with its radioluminescence spectrum (Figure S13, Supporting Information). The visible emission band is ascribed to the radiative recombination between donor and acceptor levels in the ZGO host (Figure S14, Supporting Information),^[^ ^28^ ^]^ while the NIR emission band is ascribed to the Cr^3+^ transition (^2^E → ^4^A_2_) in ZGGO (Figure S15, Supporting Information).^[^ ^29^ ^]^ The PRL intensity of ZGGO@ZGO was found to depend on the X‐ray irradiation dose, reaching full charge after 5 min of exposure (Figure 2b). After 5 min of X‐ray irradiation, the PRL of ZGGO@ZGO persisted for over 6 h (Figure 2c) and demonstrated excellent optical stability (Figure 2d). During PRL decay, the emission peak positions remained unchanged, but the relative intensity varied (Figure 2e,f). This difference is attributed to the distinct trap densities and depths associated with the visible and NIR emission bands in ZGGO@ZGO. Accordingly, two different thermoluminescence (TL) spectra were observed when monitoring the visible and NIR emissions (Figure S16, Supporting Information), each corresponding to a specific set of traps (denoted as trap 1 and trap 2 in Figure 2g). Furthermore, separate TL measurements of the individual ZGO and ZGGO phases revealed clear differences in intensity and position (Figure S17, Supporting Information). The TL spectra of single ZGO correspond to the ZGO component in core‐shell structured ZGGO@ZGO, and the TL spectra of single ZGGO correspond to the ZGGO component in core‐shell structured ZGGO@ZGO. These results further substantiate the assignment of distinct traps to the respective components in ZGGO@ZGO. Additionally, heating caused the PRL to rapidly diminish and restored ZGGO@ZGO to its initial state (Figure S18, Supporting Information), as the additional energy facilitated electron escape from the traps (Figure 2h). After repeated heating cycles, the PRL remained stable (Figure 2i), demonstrating its excellent restorability.

Based on these findings, we propose a possible PRL mechanism of ZGGO@ZGO (Figure 2j). Under X‐ray irradiation, high‐energy electrons are ejected from lattice atoms throughout ZGGO@ZGO, inducing a cascade of additional ionized electrons and generating electron‐hole pairs in both the ZGGO and ZGO, respectively (process 1), owing to their spatially separated structures. A portion of holes in the valence band (VB) of ZGGO is transitioned to the hole trap 1, and some holes in the VB of ZGO are transitioned to the hole trap 2 (process 2). Meanwhile, a portion of electrons in the conduction band (CB) of ZGGO are captured and stored in trap 1, and some of the electrons in the CB of ZGO are captured and stored in trap 2 (process 2). The captured holes and electrons stabilize the separated charges and prevent recombination. At this stage, the electrons have already undergone an initial path selection based on their captured positions. After irradiation ceases, the trapped electrons in ZGGO and ZGO are gradually released at room temperature by overcoming the respective energy barriers and return to their respective CB (process 3). Afterward, these electrons undergo non‐radiative transitions to the excited state (^4^T_2_) of Cr^3+^ in ZGGO core and the donor level in ZGO shell (process 4). Meanwhile, the captured holes in ZGGO and ZGO also transfer to the ground state of Cr^3+^ in ZGGO core and the acceptor level in ZGO shell (process 4). Finally, radiative transitions occur from the electrons in the excited state (^4^T_2_) of Cr^3+^ in ZGGO core and in the donor level in ZGO shell return to their respective holes, thereby generating their corresponding PRL (process 5).

### 2.3 Acid‐Responsive Properties of ZGGO@ZGO

**Figure 3:**
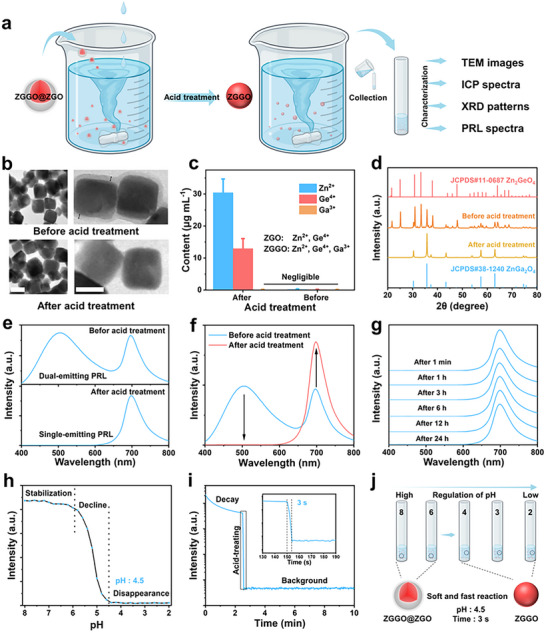
Acid‐responsive properties of ZGGO@ZGO. a) Schematic illustration of the acid‐responsive properties. b) TEM images (scale bar: 40 nm), c) ICP‐MS (*n* = 3), (d) XRD patterns, and e,f) PRL spectra of ZGGO@ZGO before and after acid treatment. g) PRL spectra of ZGGO@ZGO after acid treatment for different times. h) PRL intensity of ZGGO@ZGO at 510 nm after exposure to aqueous solution with different pH values for 10 s. i) PRL decay of ZGGO@ZGO at 510 nm during acid treatment (pH = 2). j) Schematic diagram illustrating the acid‐responsive ability of ZGGO@ZGO.

Whereafter, we investigated the acid‐responsive properties of ZGGO@ZGO via comparing TEM images, inductively coupled plasma mass spectrometry (ICP‐MS) spectra, XRD patterns, and PRL spectra before and after acid treatment (**Figure** **3**a). After acid treatment of ZGGO@ZGO, the disappearance of ZGO shell was observed (Figure 3b; Figure S19, Supporting Information). ICP‐MS analysis revealed the presence of Zn^2+^ and Ge^4+^ but the absence of Ga^3+^ in the supernatant solution of ZGGO@ZGO after acid treatment (Figure 3c), further confirming the degradation of ZGO shell and the stability of ZGGO core in acidic conditions. Additionally, the XRD peaks attributed to ZGO shell disappeared while the XRD peaks attributed to ZGGO core remained unchanged after acid treatment of ZGGO@ZGO (Figure 3d). These results indicate that the core‐shell structure of ZGGO@ZGO is disrupted after acid treatment: the ZGO shell degrades, while the ZGGO core remains intact. As expected, this structural change also alters the optical properties of ZGGO@ZGO. After acid treatment, the original dual‐emitting PRL of ZGGO@ZGO transformed into single‐emitting NIR PRL (Figure 3e). This change in the PRL spectrum affected visual observation: before acid treatment, the dual‐emitting PRL signal was visible to the naked eye (Figure S20a, Supporting Information); after acid treatment, the visible band disappeared, making the signal undetectable without instrumentation (Figure S20b, Supporting Information). However, the invisible NIR PRL signal from acid‐treated ZGGO@ZGO was detected using an NIR CCD camera (Figure S20c, Supporting Information). Such changes in optical properties provide the foundation for high‐security X‐ray imaging encryption. Furthermore, the NIR PRL intensity was enhanced after acid treatment of ZGGO@ZGO (Figure 3f; Figure S21, Supporting Information). This enhancement in the NIR PRL of ZGGO upon acid‐triggered degradation of ZGO shell can be attributed to two factors: 1) the ZGO shell may absorb some of the PRL emitted by the ZGGO core, and 2) the core‐shell interface may facilitate electron diffusion from ZGGO traps into ZGO, leading to non‐radiative transitions. Additionally, the NIR PRL of acid‐treated ZGGO@ZGO exhibited excellent optical stability (Figure S22, Supporting Information). Crucially, the PRL did not revert to its initial state after acid treatment (Figure 3g), demonstrating the irreversible acid‐responsive behavior of ZGGO@ZGO. This property enables the detection of information leakage by verifying whether the visible PRL optical information remains after X‐ray irradiation.

To further investigate the acid‐responsive behavior of ZGGO@ZGO, the visible PRL of ZGGO@ZGO was monitored after incubation in aqueous solution with different pH values. Under different pH conditions, the PRL intensity at 510 nm (representing the visible band) varied significantly (Figure 3h). The visible band intensity decreased sharply as the pH dropped from 6.0 to 4.5 and eventually disappeared. Given that many carbonated beverages have a pH below 4.0 (Figure S23, Supporting Information), the acid‐responsive behavior of ZGGO@ZGO can be easily triggered even without specialized chemical reagents such as hydrochloric acid, nitric acid, or sulfuric acid (Figure S24, Supporting Information). Moreover, the acid reaction occurred within seconds, as evidenced by the rapid decay of PRL at 510 nm during acid treatment (Figure 3i). This convenient acid‐responsive behavior and fast reaction time make ZGGO@ZGO highly suitable for practical encryption applications (Figure 3j).

### 2.4 X‐Ray Imaging Encryption

**Figure 4:**
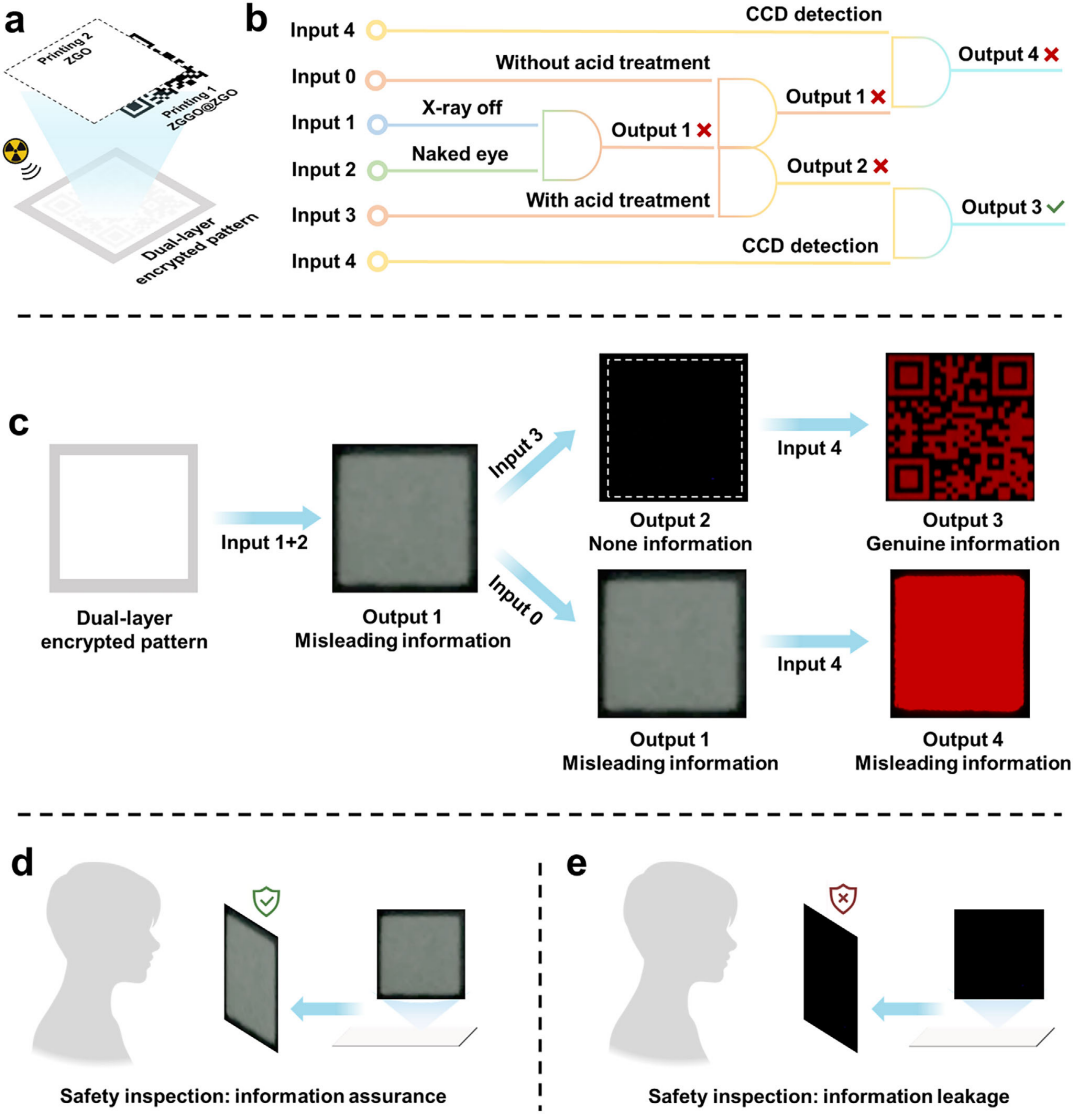
The X‐ray imaging encryption system. a) Schematic diagram of a dual‐layer encrypted pattern comprising ZGGO@ZGO and ZGO. b) Schematic diagram of sequential logic gates based on the dual‐layer encrypted pattern comprising ZGGO@ZGO and ZGO. c) Outputs of the dual‐layer encrypted pattern comprising ZGGO@ZGO and ZGO under different input conditions. d,e) Schematic illustration of the information leakage detection.

Radioluminescence memory scintillators typically emit PRL within the visible spectrum, making encrypted information directly observable to the naked eye and vulnerable to information leakage through trial‐and‐error attacks. Crucially, their PRL ultimately reverts to the initial state (complete PRL disappearance) following decryption, erasing any all traces of potential unauthorized access. This self‐erasing property presents a critical security vulnerability, as it is unable to verification of whether information leakage has occurred. In response to these challenges, we implemented an X‐ray information encryption system utilizing ZGGO@ZGO and ZGO (**Figure** **4**a; Figure S25, Supporting Information). The system was fabricated by first formulating ZGGO@ZGO into a colloidal suspension for information printing (Figure S25a, Supporting Information), followed by surface coating with a similar ZGO colloid (Figure S25b, Supporting Information) to create the final dual‐layer encrypted pattern (Figure 4a). Specifically, the dual‐layer encrypted pattern requires perfect overlap to prevent information leakage. The system incorporates multiple security features through sequential logic gates requiring acid treatment and CCD detection, coupled with deliberately engineered misleading information, effectively preventing information leakage (Figure 4b,c). Specifically, a white square pattern (output 1) was seen after X‐ray irradiation (input 1 + 2), resulting from the spectral overlap in the visible PRL bands of ZGGO@ZGO and ZGO (Figure S26, Supporting Information). The deliberate “output 1” is used as misleading information (level one), preventing the leakage of genuine information. The system maintained the same deceptive information (output 1) in the absence of acid treatment (input 0) and continued to display a similar square pattern (output 4, level two) using CCD observation (input 0+4). Correspondingly, the introduction of acid treatment (input 3) triggered the degradation of ZGO component, causing the complete disappearance of optical information to the naked eye (output 2, level three), a crucial security enhancement through information concealment. Final decryption (output 3) strictly required the sequential combination of acid treatment followed by CCD detection (input 3 + 4). The process of accessing the genuine information requires not only avoiding the triple misleading information but also performing acid treatment and CCD detection, significantly enhancing the security of the encrypted information. Crucially, the altered information became permanently unrecoverable after acid treatment due to the irreversible acid‐responsive properties of ZGGO@ZGO. This characteristic ensures reliable detection of potential information leakage during routine checks by verifying whether the visual information remains after X‐ray irradiation (Figure 4d,e).

**Figure 5:**
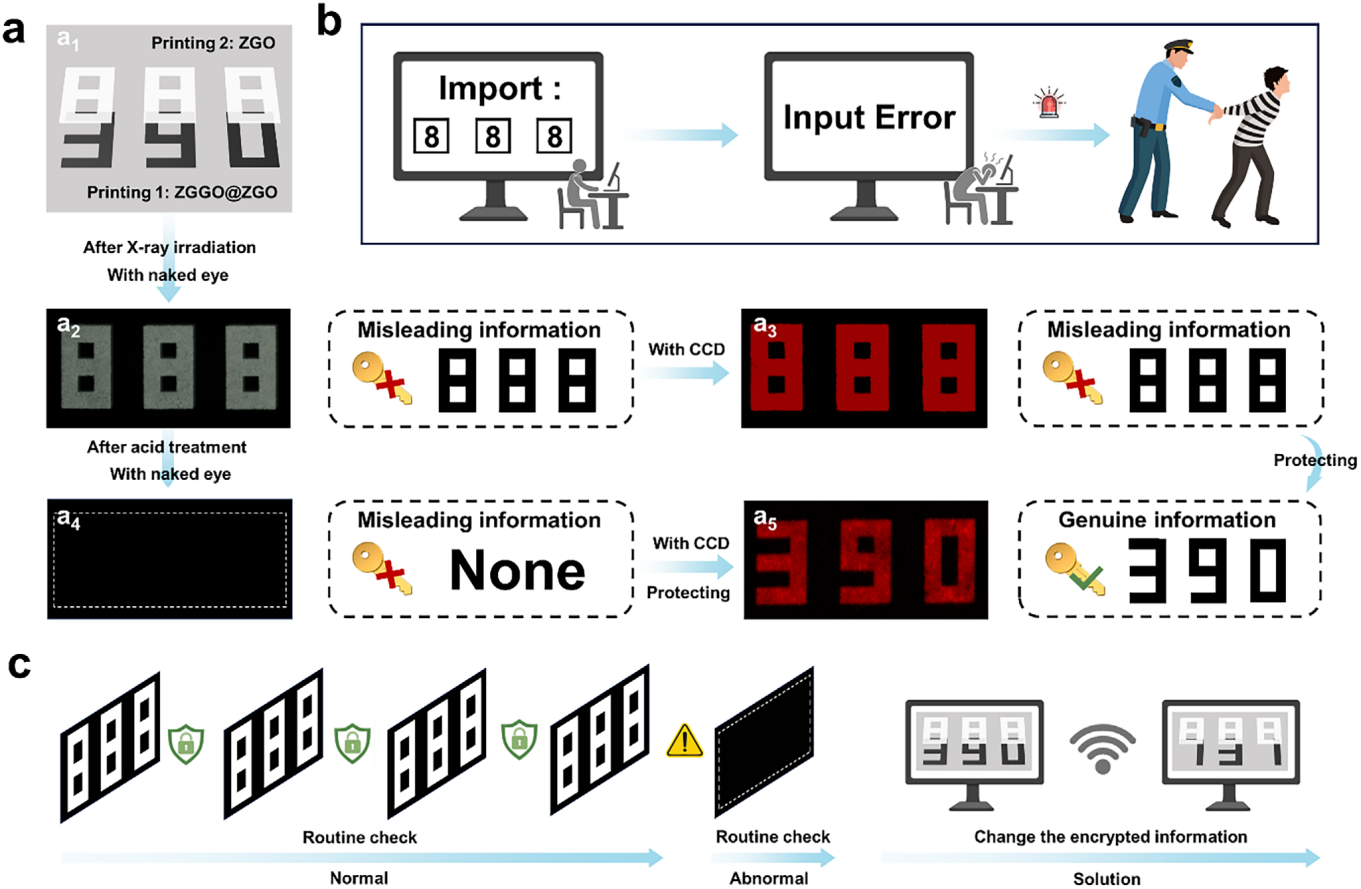
An application scenario of X‐ray imaging encryption based on the ZGGO@ZGO and ZGO system. a) The schematic diagram of a dual‐layer encrypted pattern comprising ZGGO@ZGO and ZGO, and its outputs under different input conditions. b) The schematic diagram of information protection. c) Schematic illustration of the information leakage detection and solution.

Building upon the ZGGO@ZGO and ZGO system, diverse encrypted patterns can be engineered (Figure S27, Supporting Information). Meanwhile, to demonstrate practical application, we further simulated an application scenario of X‐ray imaging encryption (**Figure** **5**). The dual‐layer encrypted pattern was composed of the figure “888” and “390” (Figure 5a_1_). After X‐ray irradiation, the figure “888” was found with the naked eye or CCD (Figures 5a_2_,a_3_), serving as an intentional decoy designed to mislead potential offenders. This strategic design induces offenders to mistake the visible “888” for genuine information, potentially causing them to abandon further decryption attempts. Should offenders attempt to use this false information, the security system can promptly identify and apprehend them (Figure 5b). This misleading information not only helps to prevent the leakage of genuine information but also has the potential to arrest offenders. For more sophisticated offenders attempting acid treatment to reveal hidden information, the system maintained robust protection, as they failed to extract any visually discernible information with the naked eye (Figure 5a_4_). This makes offenders who conduct acid treatment think that the information has been corrupted and disappeared, leading them to abandon further decryption attempts. The hidden information just came out with CCD detection after acid treatment (Figure 5a_5_). Furthermore, even if information leakage occurs, a routine check can detect the breach by monitoring whether the visual information remains after X‐ray irradiation (Figure 5c). By promptly updating the encrypted information and notifying relevant stakeholders, the impact of information leakage can be minimized. Collectively, this high‐security encryption approach not only safeguards hidden information through deception mechanisms but also enhances traceability and response capabilities against information leakage.

## 3 Conclusion

This study presents a high‐security X‐ray imaging encryption technology that utilizes an irreversible acid‐responsive radioluminescence memory scintillator (ZGGO@ZGO) for addressing the critical issues of information leakage and undetectable breaches in traditional information encryption systems. Specifically, the ZGGO@ZGO exhibits dual‐emitting PRL in visible and NIR regions, with the visible light acting as a decoy to mislead offenders. Upon acid treatment, the dual‐emitting PRL of ZGGO@ZGO is irreversibly transformed into to single‐emitting NIR PRL. The acid‐responsive optical change is attributed to the degradation of ZGO. Specifically, acids stronger than H_4_GeO_4_ can displace the ${\mathrm{GeO}}_{4}^{4-}$ in Zn_2_GeO_4_ to generate the degradation of shell‐ZGO and quench the visible PRL. Meanwhile, the remaining NIR PRL can only be captured using a CCD camera. The dual‐layer security pattern composed of ZGGO@ZGO and ZGO, integrating visual misdirection with acid treatment and CCD detection sequential logic gates, significantly reduces the possibility of information leakage. Crucially, the irreversible nature of PRL transformation ensures that any breach is immediately detectable by monitoring whether the visual information remains after X‐ray irradiation, enabling prompt risk mitigation. This approach not only significantly improves information security but also overcomes the limitations of the inability to real‐time leakage detection in conventional encryption systems, offering new perspectives for information protection in specific fields such as public livelihood and national security.

## 4 Experimental Section

### Chemicals

Na_2_GeO_3_ (99.99%), Eu(NO_3_)_3_·6H_2_O (99.99%), Ga(NO_3_)_3_·xH_2_O (99.99%), Zn(NO_3_)_2_·xH_2_O (99.99%), Cr(NO_3_)_3_·xH_2_O (99.99%), GeO_2_ (99.99%), NaOH (99.9%), NH_3_·H_2_O (25‐28%), C_6_H_8_O_7_·H_2_O (99.8%) Polyvinyl alcohol (PVA), and acetic acid were afforded by Aladdin‐Reagent Co. Ltd (China).

### Instruments

A fluorescence spectrometer (FLS920, Edinburgh Instruments, UK), equipped with 450 W xenon lamp, X‐ray generator (50 kV, 80 µA), and R928 photomultiplier tube, was used to acquire radioluminescence and photoluminescence spectra, persistent radioluminescence (PRL) spectra, and decay curves. Thermoluminescence spectra were obtained using a DSC600 temperature‐controlled stage (Linkam, UK). A MiniFlex 600 X‐ray diffractometer (Rigaku, Japan) with a Cu Kα X‐ray tube was used to acquire the X‐ray diffraction (XRD) patterns. Transmission electron microscopy (TEM) and high‐resolution TEM (HR‐TEM) images, EDS spectra, and corresponding mapping images were a Hitachi H7650 system (Hitachi Co., Ltd., Tokyo, Japan) equipped with an energy‐dispersive X‐ray spectroscope. X‐ray photoelectron spectroscopy (XPS) was performed using an Axis Supra XPS surface analysis instrument (Kratos, UK). The elemental contents were determined using an inductively coupled plasma optical emission spectrometer (ICP‐OES, ULTIMA 2, HORIBA Scientific Co., Ltd., Kyoto, Japan).

### Synthesis of ZGGO@ZGO

First, Zn_1+x_Ga_2‐2x_Ge_x_O_4_:Cr^3+^ (x = 0–0.4) PLNPs were prepared via the hydrothermal method. Briefly, Zn(NO_3_)_2_ solution (1 mol/L), Ga(NO_3_)_2_ solution (1 mol/L), Cr(NO_3_)_2_ solution (0.1 mol/L), and Ge solution (0.25 mol/L) were added into the aqueous solution (11 mL) according to the stoichiometric ratio. Then add NH_3_·H_2_O to bring the pH of the system to 9 and stir at room temperature for 1 h. After stirring, the system was transferred to reactor heating at 220 °C for 10 h. Finally, the obtained particle was centrifuged and dried for use and characterization.

After obtaining ZGGO, ZGO was coated on the surface of ZGGO by the sol‐gel method. Briefly, Eu(NO_3_)_3_ (0.1 mol/L), Ge solution (0.25 mol/L), Zn(NO_3_)_2_ solution (1 mol/L), and 1.2608 g C_6_H_8_O_7_·H_2_O were added into the aqueous solution (15 mL) according to the stoichiometric ratio. Then the pH of the system was adjusted to 6.0 by NH_3_·xH_2_O and stirring at room temperature for 1 h. The equivalent mass ZGGO in 10 mL aqueous solution was added into the system and ultrasound for 15 min. The mixed system was heated at 75 °C for gel formation under stirring. The gel system was heated at 130 °C for 3 h and then at 200 °C for 7 h. Finally, the obtained fluffy materials were heated at 800 °C for 3 h to obtain ZGGO@ZGO.

### Preparation of Encryption Patterns

All of the encryption patterns were prepared using the screen‐printing technique; the sticky phosphor slurry was formed via evenly mixing PVA with materials (ZGGO@ZGO or ZGO) to prepare the encryption patterns. The visible optical images were collected using an iPhone 12, and the invisible optical images were collected using a BR‐DD camera (Photometrics, Canada).

### Statistical Analysis

The experimental results were expressed as mean standard deviation by Origin 2021.

## Conflict of Interest

The authors declare no conflict of interest.

## Supporting information



Supporting Information

## Data Availability

The data that support the findings of this study are available from the corresponding author upon reasonable request.
