# Supplementary material for: High‐Security X‐Ray Imaging Encryption Based on Irreversible Acid‐Responsive Radioluminescence Memory Scintillator
Source: Adv Sci (Weinh). 2025 Dec 17;13(12):e20158. doi: 10.1002/advs.202520158 (PMC12948202; doi:10.1002/advs.202520158)
Supplement: Supplementary file 1 — Supporting Information [file ADVS-13-e20158-s001.docx]

Supporting Information

High-Security X-ray Imaging Encryption Based on Irreversible Acid-Responsive Radioluminescence Memory Scintillator

*Lin Liu^+^, Shanshan Peng^+^, Hanjing Guo, Linping He, Xiaofang Luo, Zixuan Li, Lisen Lin* and Huanghao Yang**

L. Liu, H. Guo, L. He

Department of Nuclear Medicine, the First Affiliated Hospital of Fujian Medical University, Fujian Medical University, Fuzhou 350005, China

S. Peng, X. Luo, Z. Li, Prof. L. Lin, Prof. H. Yang

New Cornerstone Science Laboratory, MOE Key Laboratory for Analytical Science of Food Safety and Biology, College of Chemistry, Fuzhou University, Fuzhou 350108, China

E-mail: lisen.lin@fzu.edu.cn; hhyang@fzu.edu.cn

Funding: The National Key Research and Development Program of China (2020YFA0210800), the National Natural Science Foundation of China (22374026 and 22027805), the Major Project of Science and Technology of Fujian Province (2020HZ06006) and the Natural Science Foundation of Fujian Province (2025J08172).

Keywords: radioluminescence memory scintillator, X-ray imaging encryption, persistent radioluminescence, high-security, acid-responsiveness


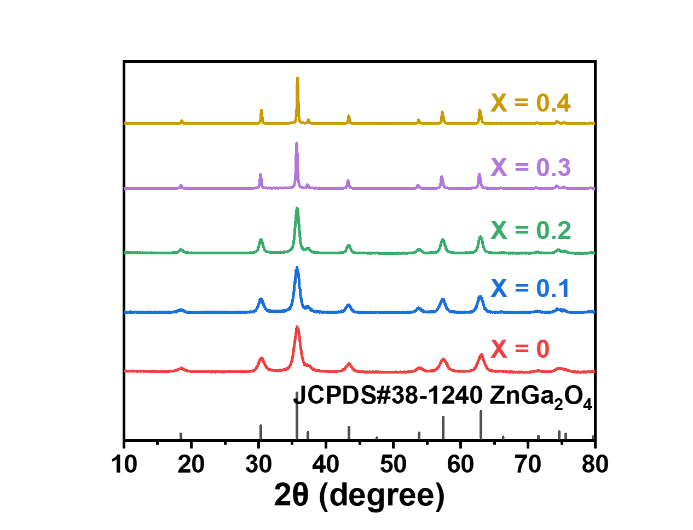


**Figure S1.** XRD patterns of Zn_1+x_Ga_2-2x_Ge_x_O_4_:Cr^3+^ (X = 0-0.4).


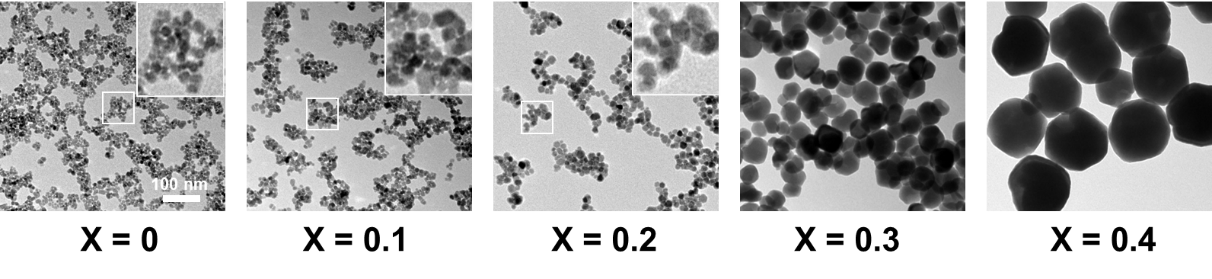


**Figure S2.** TEM images of Zn_1+x_Ga_2-2x_Ge_x_O_4_:Cr^3+^ (X = 0-0.4).


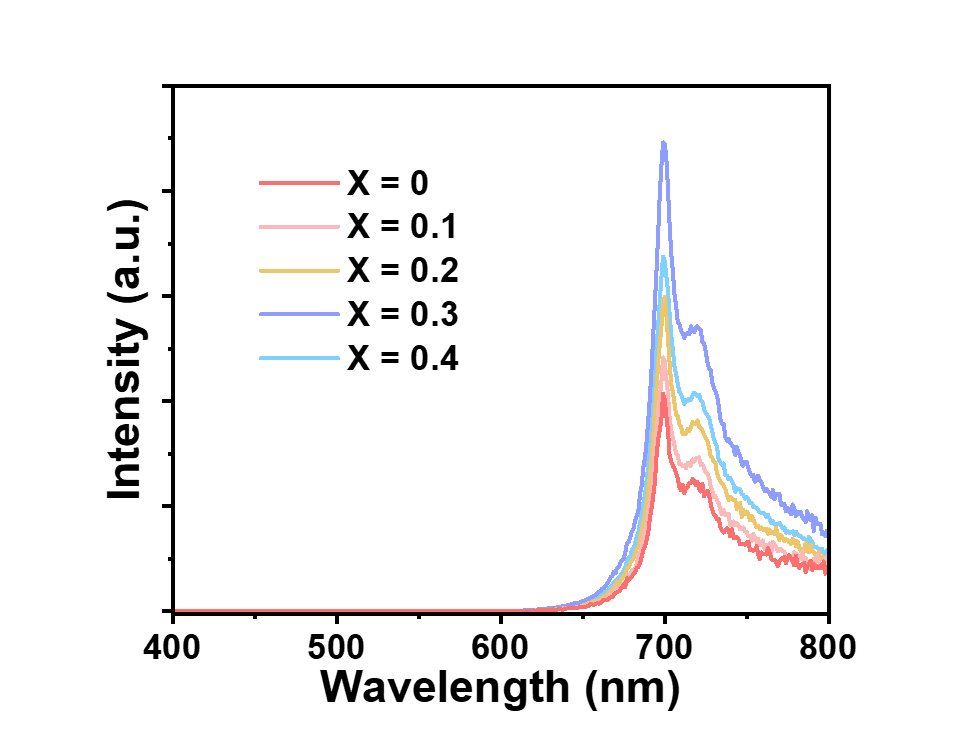


**Figure S3.** PRL spectra of Zn_1+x_Ga_2-2x_Ge_x_O_4_:Cr^3+^ (X = 0-0.4) after X-ray irradiation for 5 min.


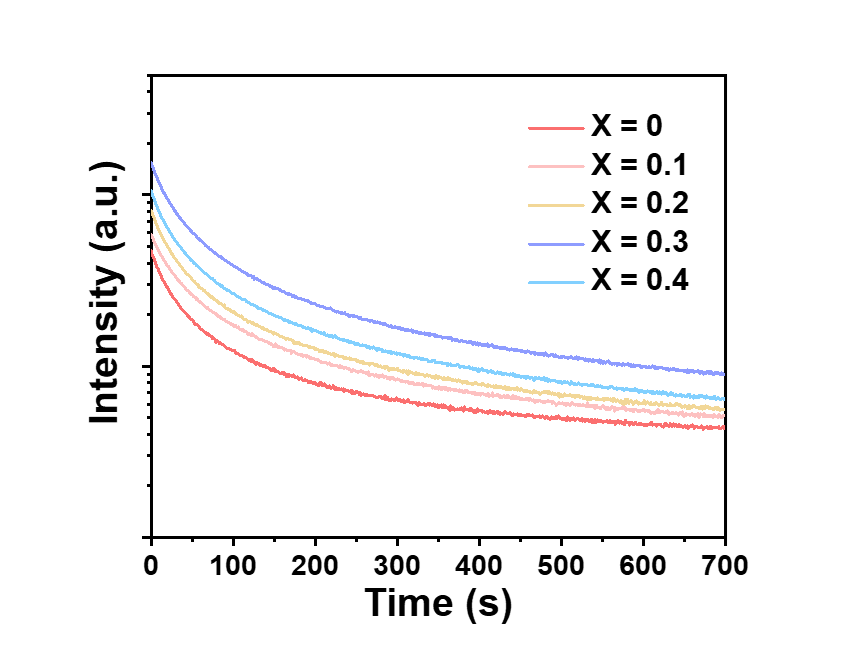


**Figure S4.** PRL decay of Zn_1+x_Ga_2-2x_Ge_x_O_4_:Cr^3+^ (X = 0-0.4) after X-ray irradiation for 5 min.


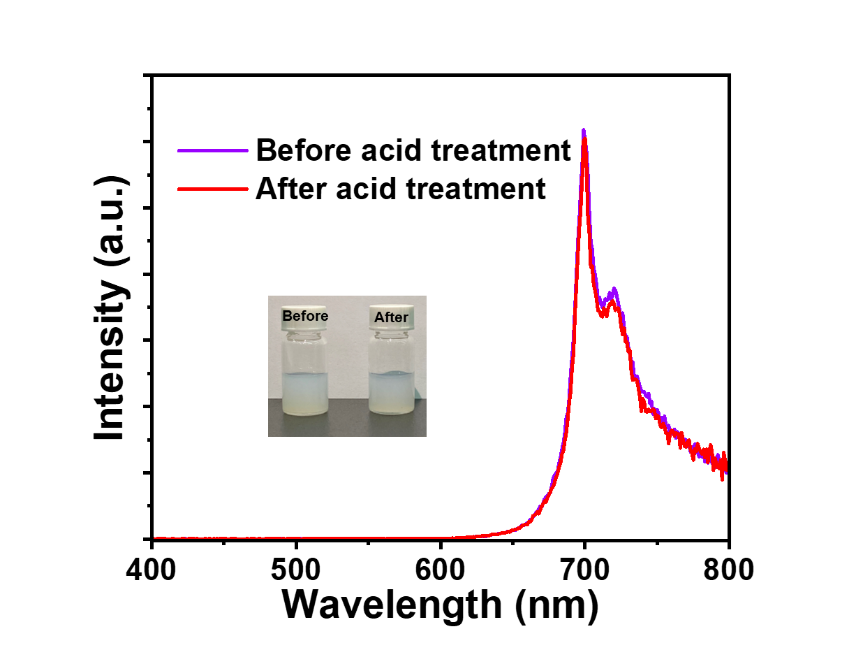


**Figure S5.** ZGGO dispersed in aqueous solution images and its PRL spectra before/after acid treatment.


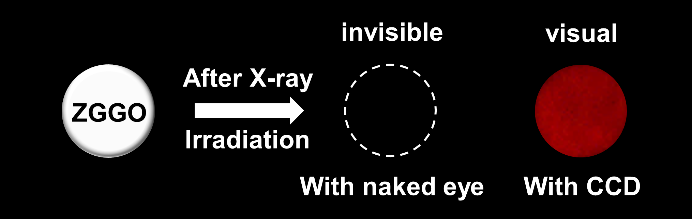


**Figure S6.** ZGGO images with naked eye or CCD images after X-ray irradiation.


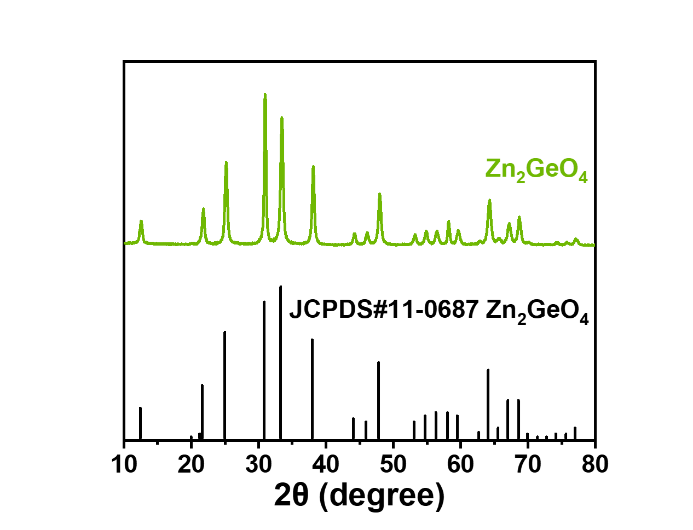


**Figure S7.** XRD pattern of Zn_2_GeO_4_.


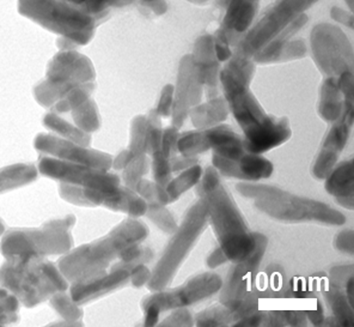


**Figure S8.** TEM image of Zn_2_GeO_4_.


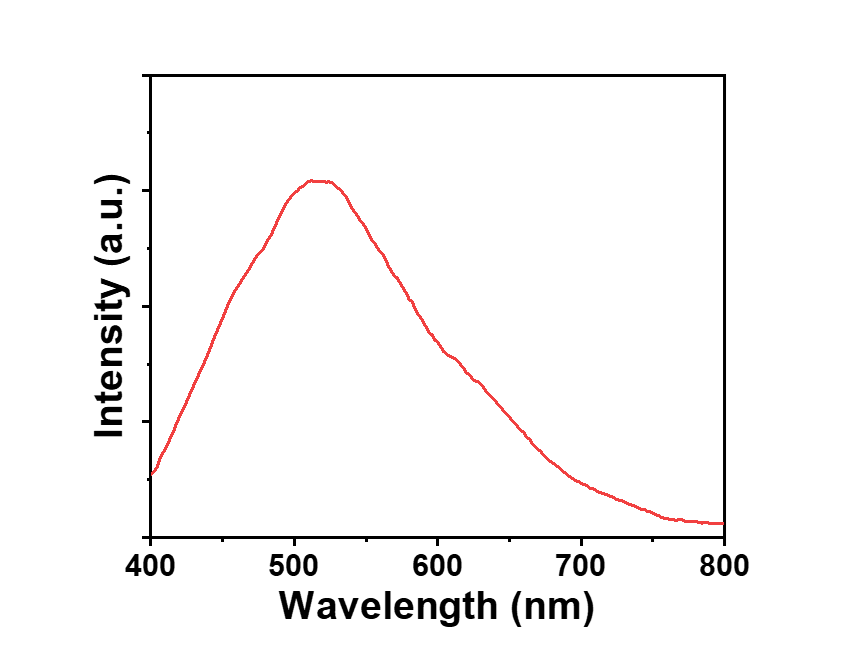


**Figure S9.** PRL spectrum of Zn_2_GeO_4_ after X-ray irradiation for 5 min.


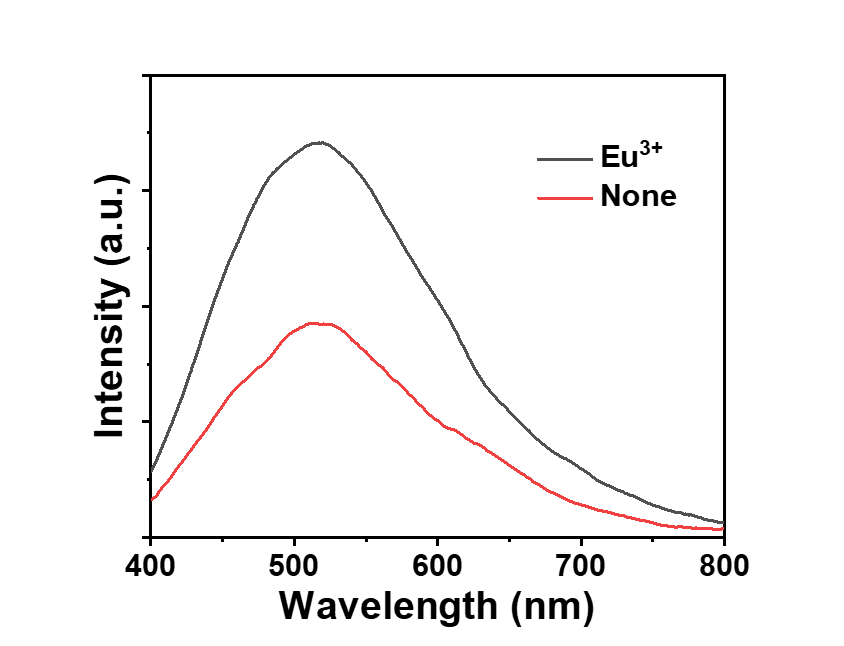


**Figure S10.** PRL spectra of Zn_2_GeO_4_ with or without Eu^3+^ doping.


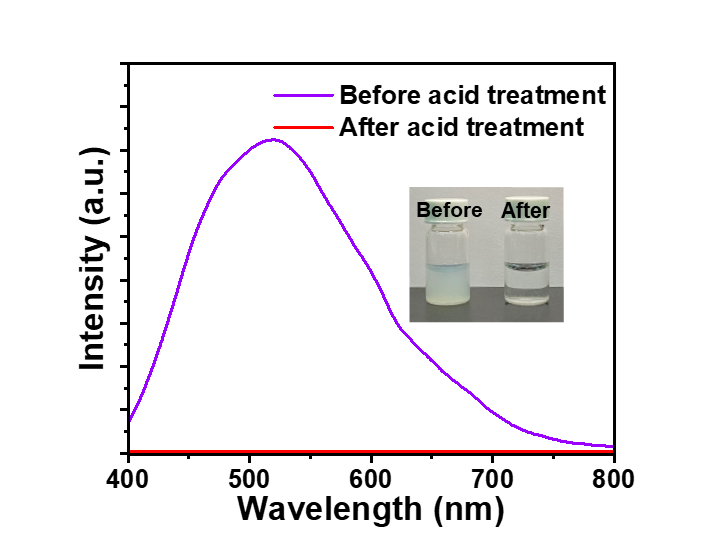


**Figure S11.** ZGO dispersed in aqueous solution image and its PRL spectra before/after acid treatment.


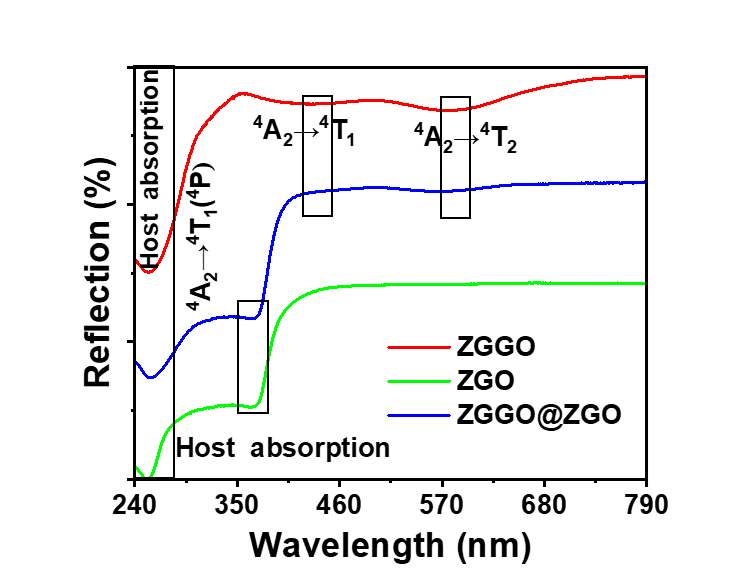


**Figure S12.** DRS of ZGGO, ZGO and ZGGO@ZGO.


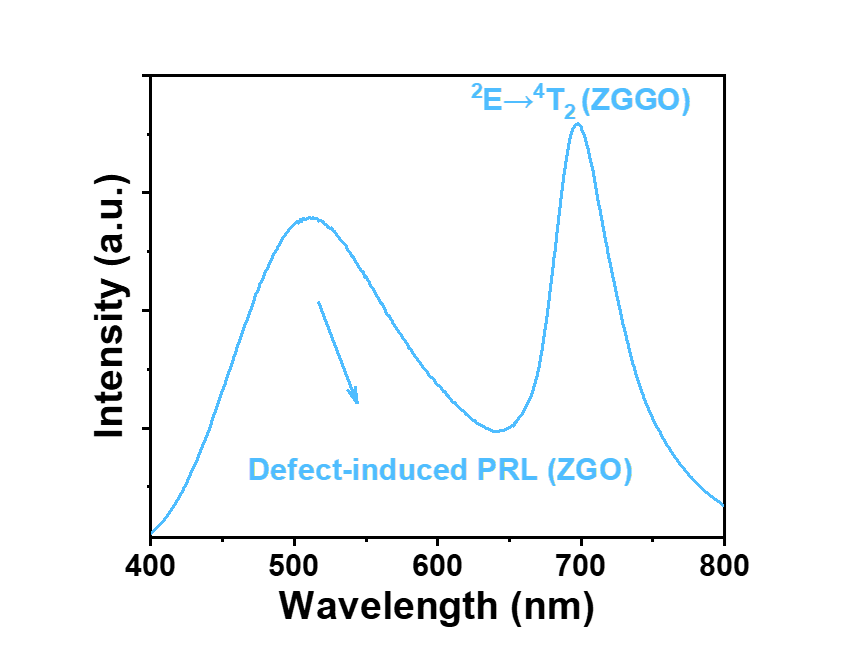


**Figure S13.** Radioluminescence spectrum of ZGGO@ZGO.


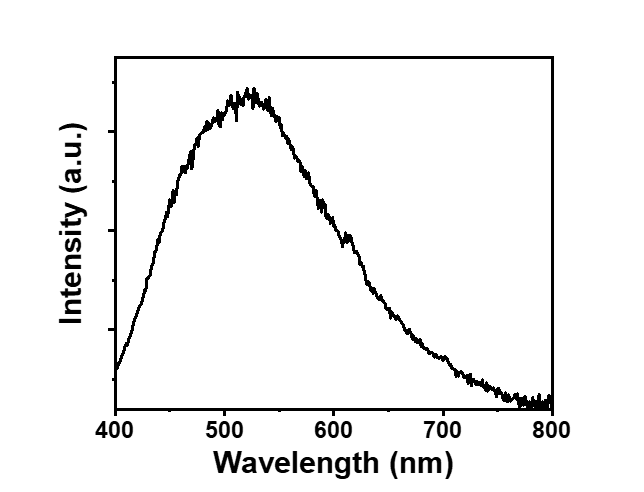


**Figure S14.** Radioluminescence spectrum of ZGO.


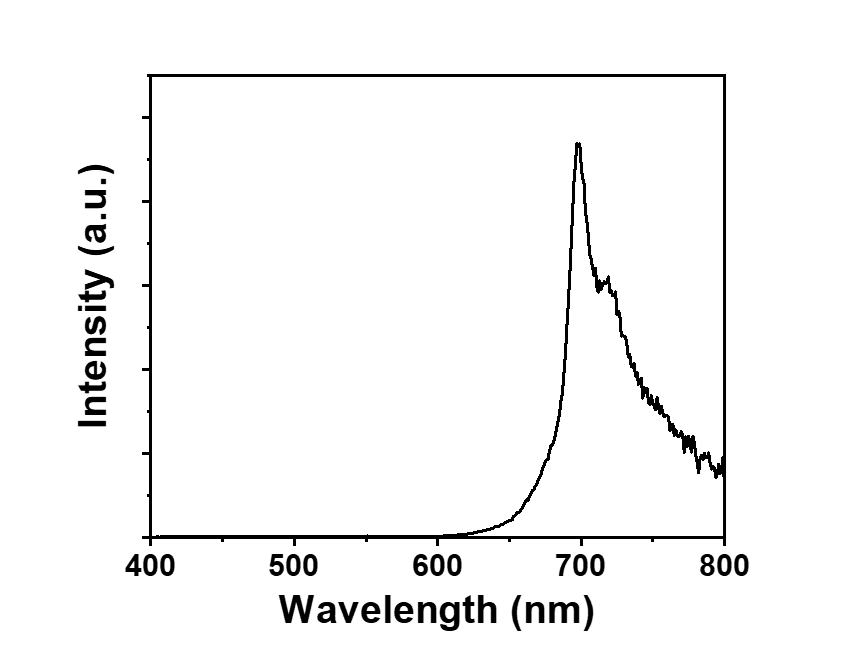


**Figure S15.** Radioluminescence spectrum of ZGGO.


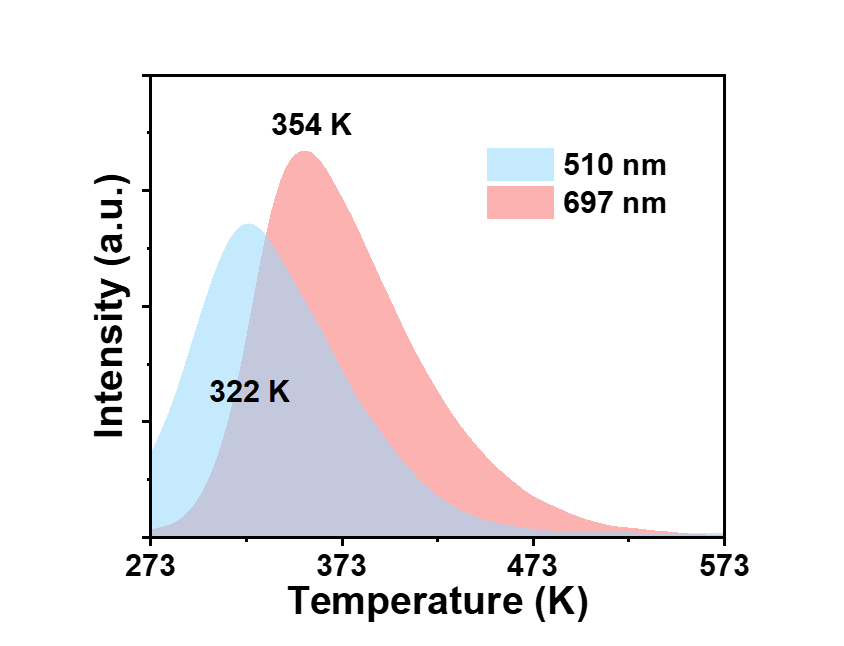


**Figure S16.** Thermoluminescence spectra of ZGGO@ZGO after X-ray irradiation for 5 min.


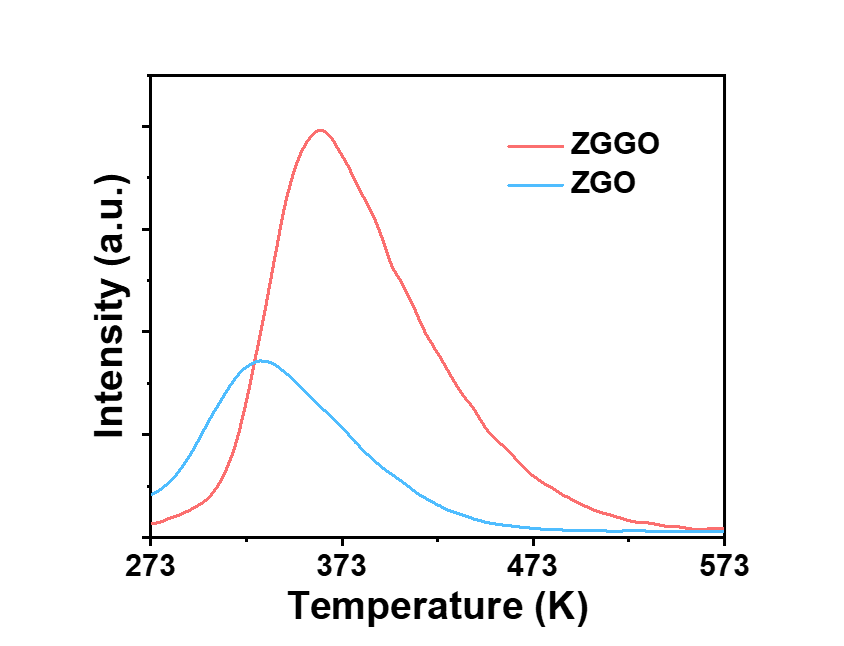


**Figure S17.** Thermoluminescence spectra of ZGO and ZGGO after X-ray irradiation for 5 min.


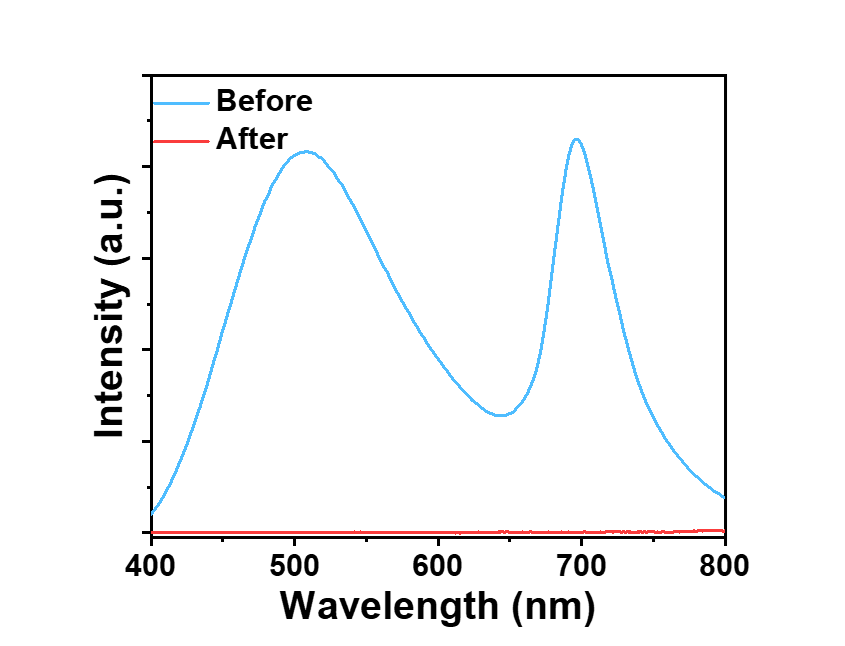


**Figure S18.** PRL spectra of ZGGO@ZGO before/after heating at 150 °C for 15 min.


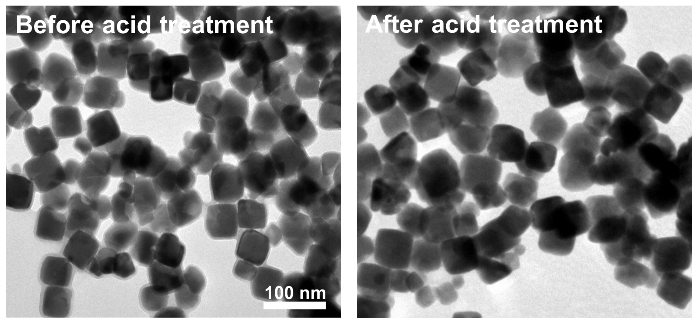


**Figure S19.** TEM images of ZGGO@ZGO before/after acid treatment (Figure 3b is obtained by enlarging these images).


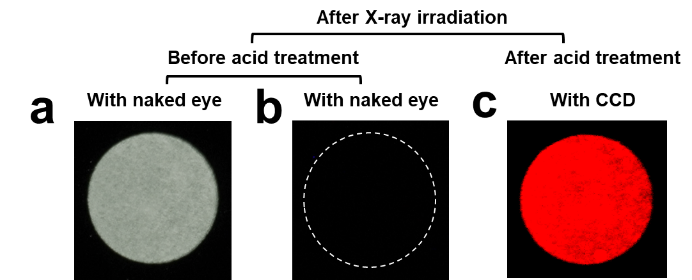


**Figure S20.** ZGGO@ZGO optical images after X-ray irradiation with naked eye (a) before/ (b) after acid treatment, and (c) ZGGO@ZGO images with CCD camera acid treatment.


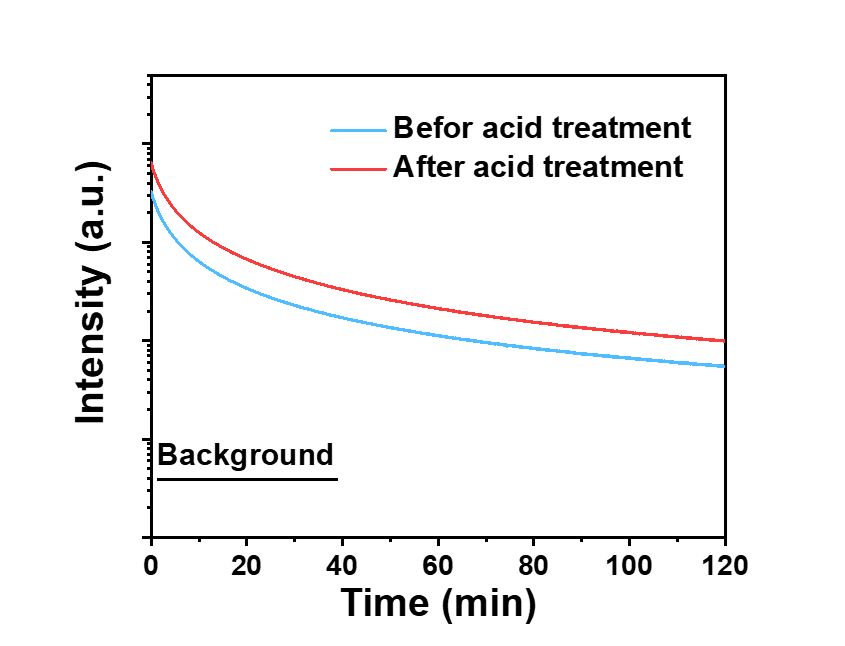


**Figure S21.** PRL decay at 697 nm of ZGGO@ZGO before/after acid treatment.


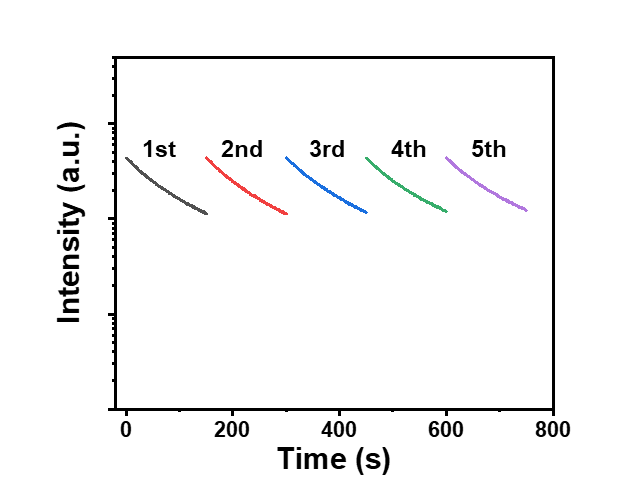


**Figure S22.** PRL decay at 697 nm of acid-treating ZGGO@ZGO with multiple X-ray irradiations for 5 min.


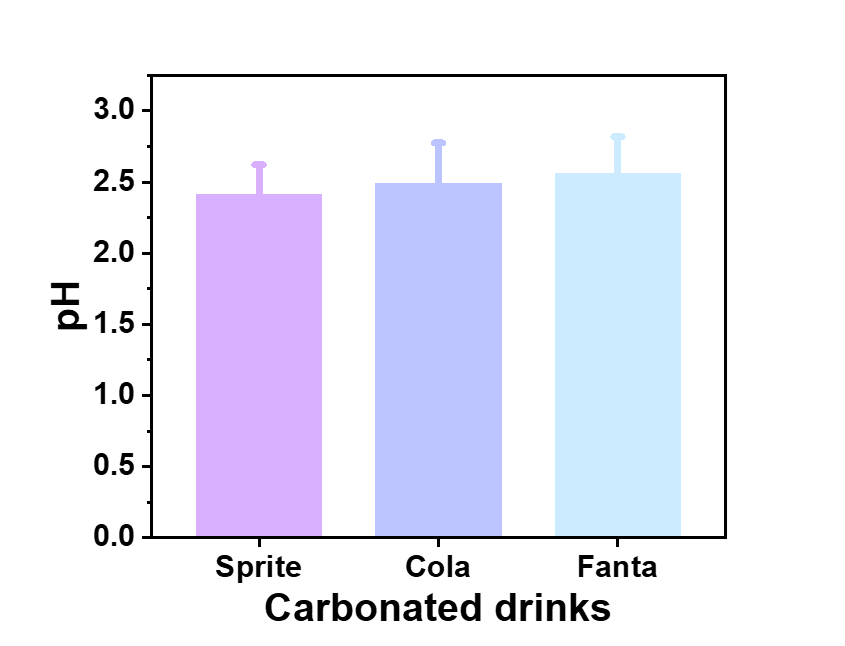


**Figure S23.** pH value of different carbonated drinks.

**
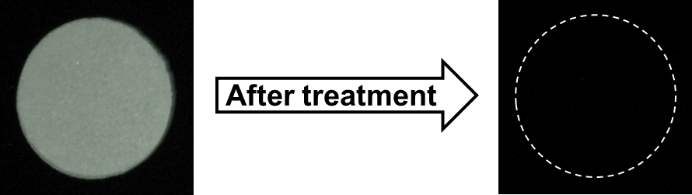
**

**Figure S24.** Visual images of ZGGO@ZGO before/after carbonated drink (sprite) treatment.


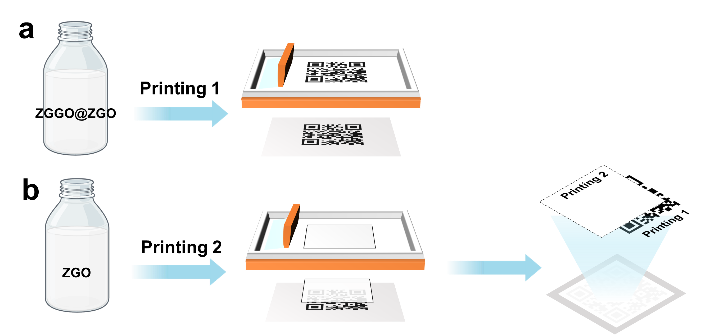


**Figure S25.** The preparation process of composite pattern composed of ZGGO@ZGO and ZGO.


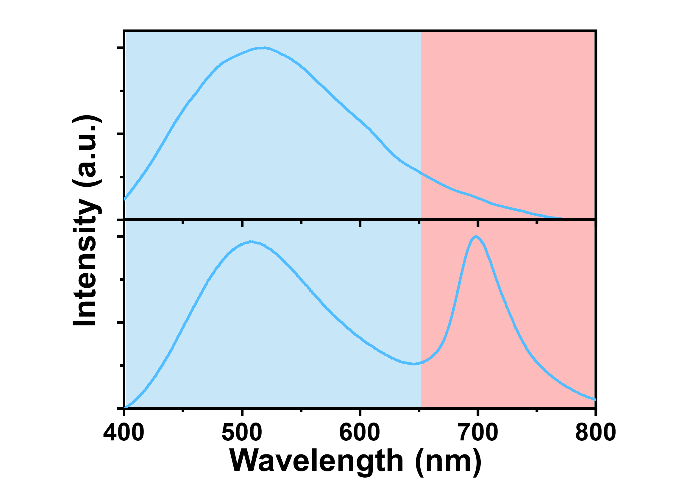


**Figure S26.** PRL spectra of ZGO and ZGGO@ZGO after X-ray irradiation for 5 min.


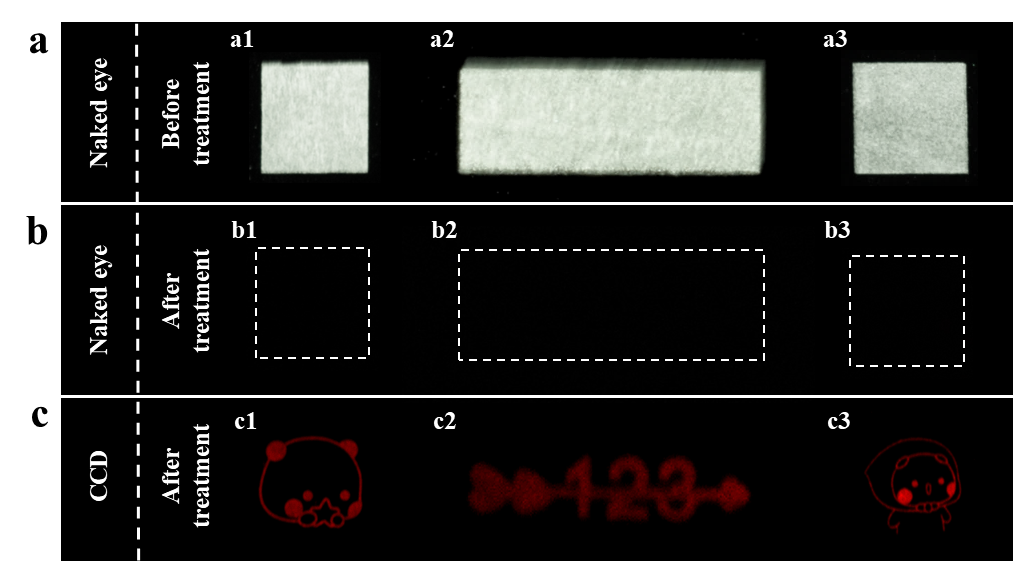


**Figure S27.** The diverse encrypted patterns composed of ZGGO@ZGO and ZGO.
